# The Alignment of Dietary Intake and Symptom-Reporting Capture Periods in Studies Assessing Associations between Food and Functional Gastrointestinal Disorder Symptoms: A Systematic Review

**DOI:** 10.3390/nu11112590

**Published:** 2019-10-28

**Authors:** Kerith Duncanson, Tracy Burrows, Simon Keely, Michael Potter, Gayatri Das, Marjorie Walker, Nicholas J. Talley

**Affiliations:** 1 School of Health Sciences, Faculty of Health and Medicine, The University of Newcastle, Newcastle, NSW 2308, Australia; tracy.burrows@newcastle.edu.au; 2 Priority Research Centre in Physical Activity and Nutrition, The University of Newcastle, Newcastle, NSW 2308, Australia; 3 Priority Research Centre, Digestive Health and Neurogastroenterology, The University of Newcastle, Newcastle, NSW 2308, Australia; simon.keely@newcastle.edu.au (S.K.); michael.potter@newcastle.edu.au (M.P.); gayatri.das@uon.edu.au (G.D.); marjorie.walker@newcastle.edu.au (M.W.); nicholas.talley@newcastle.edu.au (N.J.T.); 4 School of Biomedical Sciences and Pharmacy, The University of Newcastle, Newcastle, NSW 2308, Australia; 5 School of Medicine and Public Health, The University of Newcastle, Newcastle, NSW 2308, Australia

**Keywords:** dietary assessment, irritable bowel syndrome, functional dyspepsia, functional gastrointestinal disorder, systematic review

## Abstract

Food ingestion is heavily implicated in inducing symptoms of irritable bowel syndrome (IBS) and functional dyspepsia (FD), which affect over one-third of adults in developed countries. The primary aim of this paper was to assess the alignment of dietary assessment and symptom-reporting capture periods in diet-related studies on IBS or FD in adults. Secondary aims were to compare the degree of alignment, validity of symptom-reporting tools and reported significant associations between food ingestion and symptoms. A five-database systematic literature search resulted in 40 included studies, from which data were extracted and collated. The food/diet and symptom capture periods matched exactly in 60% (*n* = 24/40) of studies, overlapped in 30% (*n* = 12/40) of studies and were not aligned in 10% (*n* = 4/40) of studies. Only 30% (*n* = 12/40) of studies that reported a significant association between food and global gastrointestinal symptoms used a validated symptom-reporting tool. Of the thirty (75%) studies that reported at least one significant association between individual gastrointestinal symptoms and dietary intake, only four (13%) used a validated symptom tool. Guidelines to ensure that validated symptom-reporting tools are matched with fit-for-purpose dietary assessment methods are needed to minimise discrepancies in the alignment of food and symptom tools, in order to progress functional gastrointestinal disorder research.

## 1. Introduction

More than one-third of adults in developed countries have chronic unexplained gastrointestinal (GI) symptoms that are classified as a functional GI disorder (FGID) [1,2]. FGIDs are characterised by the absence of any structural or biochemical explanation for gastrointestinal symptoms [3,4]. Despite the high prevalence and characteristic features, the etiopathogenesis of FGIDs are poorly understood, and there are no existing objective diagnostic tests. Irritable bowel syndrome (IBS) and functional dyspepsia (FD) are the most prevalent FGIDs [5,6]. Although these conditions are highly heterogeneous, IBS can be subtyped into constipation-dominant IBS (IBS-C), diarrhoea-dominant IBS (IBS-D), IBS with mixed bowel habits (IBS-M) and unclassified IBS (IBS-U) [3,7] and FD is classified as either Postprandial Distress Syndrome (PDS) or Epigastric Pain Syndrome (EPS), with a high degree of overlap between subgroups. In all cases, diagnosis is reliant on a patient-reported rating of gut symptoms, which do not account for associated extra-intestinal symptoms such as anxiety, depression and fatigue [4,8].

To measure changes in symptoms, FGID research relies on clinician or researcher-reported change on a symptom scale or a patient-defined judgment of improvement [9]. Outcome assessment tools in FGID treatment trials can be broadly categorised as a global scale, a validated symptom questionnaire, or a quality of life instrument [10]. The nature of the outcome of interest influences the duration and method of data capture, and level and type of validation [10]. Validation studies of these instruments involve tools that specify a timeframe or capture period for symptom reporting. For example, the IBS Severity Scoring System (IBS-SSS) is validated for assessing global symptomology over the previous ten days and the “adequate relief” question, validated for use over the previous week. For individual symptom assessment, the Bristol Stool Form (BSF) is a validated tool, but individual symptoms are usually contained within the global symptomology tools, but are not validated individually [10].

Although IBS and FD induce symptoms in different parts of the digestive tract, dietary intake is implicated in both the induction and management of symptoms of both FGIDs [11,12]. By definition and diagnosis, the symptoms of PDS subtype FD are evident after eating or drinking, whereas EPS symptoms may arise after eating. This may account for the overlap observed between EPS and PDS subtypes of FD [13]. Intervention studies highlight the association of FD symptoms with eating, without necessarily determining whether eating itself or specific dietary components are implicated. In one study of 218 patients, an increase in the intensity of FD symptoms was reported within fifteen minutes after eating and remained elevated for at least four hours [14]. In another study involving 67 IBS patients and 16 healthy adult controls, significant associations between time and postprandial IBS symptoms were reported at between 30 minutes and 4 hours after eating [15].

Gastrointestinal motility, visceral sensitivity, immune regulation, inflammation, gut barrier function and microbiome can all be influenced by dietary intake [3]. Each of these factors, individually, or in combination with intraluminal diet-related factors, have been implicated in the pathogenesis of FGIDs [3]. Dietary factors investigated in relation to IBS often include fermentable carbohydrates (FODMAPs), milk and dairy, dietary fibre, gluten, probiotics and food chemical hypersensitivity [16]. Foods high in dietary fats, wheat, caffeine or alcohol have been also implicated in FD symptom induction [11].

Assessing food and nutrient intakes is critical to understanding diet–disease relationships and managing diet-related components of health conditions [17]. Accurate dietary assessment is complex and challenging, particularly in the rapidly evolving global food environment [17]. A range of validated dietary assessment methods exist for quantitative assessment of dietary intake, and they can be broadly classified as either retrospective (past intake) or prospective (current intake). Each particular dietary assessment method has its own benefits and limitations and is suited to different purposes. A major limitation that is common to most dietary assessment methods is the inaccuracy inherent in self-report data. The nature and extent of these limitations vary depending on whether data is derived from a traditional or newer, technology-based method [17]. Validation of dietary assessment methods is not universal, as the validation may be specific to a particular population group and may have been conducted using a reference method that is objective (criterion validity) or compared with an existing validated method (relative validity). A further limitation in the field of gastrointestinal research is that food composition databases do not routinely contain data on the food components of interest, for example gluten and fermentable carbohydrates.

The assessment methods most commonly used to prospectively examine dietary intake are 24 hour (h) recalls, weighed food records and estimated food records. Diet histories, food-frequency questionnaires (FFQ) and short assessment screeners are the main methods of examining intake retrospectively. Within each method, considerable variation exists depending on whether the validated tool has been modified for use in the population group, the frequency and duration of implementation, and the skills and experience of researchers [18]. The cost, time and burden to participants and researchers of the more specific, current or “real-time” methods can be prohibitive [18] and thus retrospective dietary assessment methods are often more feasible, but the capture period may be inappropriate for the study hypothesis and research question. For example, a food frequency questionnaire that assesses usual intake over the previous three months is not suitable to investigate associations between dietary intake and postprandial gastrointestinal symptoms immediately after eating.

Given these potential inadequacies, the aim of this systematic review was to investigate dietary assessment methodology in functional gastrointestinal research by critically assessing the alignment of the dietary assessment capture period and FGID symptom-reporting period in studies that examined relationships and reported on significant associations between dietary intake and FD or IBS symptoms. 

## 2. Material and Method

### 2.1. Search Strategy

A systematic literature search was conducted in five databases (MEDLINE, Medline in process, EMBASE, PsycINFO and the Cochrane central register of controlled trials) for studies that examined associations between FGID symptoms and food or food components in adults (18 years and older). The search terms related to the following three domains (i) symptoms of functional dyspepsia OR irritable bowel syndrome OR presumed gastroduodenal symptoms (e.g., epigastric pain, early satiety, postprandial fullness, belching, nausea or vomiting, diarrhoea and constipation) and (ii) a dietary factor, nutrient, food or food component and (iii) a dietary assessment method. The search strategy is described in detail in Supplementary Table S1. The search was restricted to English language, human studies and timeframe limited from January 2000 to April 2019.

### 2.2. Primary and Secondary Outcomes

The primary outcome was the alignment of the symptom-reporting timeframe and the dietary intake capture period of the dietary assessment tool. The secondary outcome was the proportions of studies with full, partial or no overlap between dietary intake and symptom assessment that reported significant associations between food ingestion and FGID symptoms. The participants, interventions, comparisons, outcomes and study design (PICOS) used to systematically review relationship between food ingestion and symptom reporting in functional gastrointestinal disorders is shown in Supplementary Table S2.

### 2.3. Inclusion and Exclusion Criteria

#### 2.3.1. Inclusion Criteria

Studies were included if (i) participants were adults (18 years or older) who were reported as being diagnosed with IBS or FD using Rome criteria (I or II or III or IV) and had a negative upper endoscopy or colonoscopy and (ii) the paper reported on at least one FGID (functional dyspepsia and irritable bowel syndrome) symptom and (iii) the paper reported on a dietary factor, either nutrient, food or food component and the method of assessing the dietary factor and (iv) the paper reported on the relationship between at least one dietary intake factor and at least one FGID symptom. The review considered experimental and epidemiological study designs including randomized controlled trials, non-randomized controlled trials, pre–post studies, prospective and retrospective cohort studies, case control studies and analytical cross-sectional studies.

#### 2.3.2. Exclusion Criteria

Studies were excluded if participants were children or adolescents under 18 years old, if the ROME criteria was not reported as being used for diagnosis of the FGID, if they did not exclude participants who had a positive upper endoscopy for FD (to excluded peptic ulcer, oesophagitis, coeliac disease or cancer) or colonoscopy for IBS (to rule out the presence of inflammatory bowel disease or cancer) or did not have a separate study arm for participants with only FGID diagnosis. Studies were excluded if they reported on eating but did not report on a dietary factor and the relationship between the dietary factor/s and FGID symptoms. Study types that were excluded were letters to editor, conference abstracts, reviews, case reports, and research theses.

### 2.4. Data Extraction

Data extraction and reporting of results was conducted in accordance with the Preferred Reporting Items for Systematic Reviews and Meta-Analyses (PRISMA) checklist [19] (Supplementary Table S3). All studies identified in the database searches were retrieved, consolidated, screened for duplication and then assessed for inclusion using Covidence systematic review software (Veritas Health Innovation, Melbourne, Australia: available at www.covidence.org). Two reviewers independently assessed relevance for inclusion by screening titles and abstracts against inclusion criteria (GD and MP). Conflicts were resolved by a third reviewer and full texts of included abstracts were retrieved (KD). The full texts of retrieved studies were subsequently assessed by two independent reviewers (KD and GD) to determine whether they met inclusion criteria. Conflicts were resolved by a third reviewer (TB).

Data were extracted from included studies using a purpose-built excel spreadsheet tool developed by the authors, with the following variables extracted: study location (Country); study design; year and duration of study; participant characteristics (e.g., age, gender); dietary assessment method/s; dietary outcomes; food intake/consumption or administration method or variable; FGID diagnosis; FGID symptoms, FGID symptom-reporting tool or method. The tool was piloted on the first five studies, with minor amendments made before finalising extraction of data from all included studies.

The dietary instrument/s reported were extracted into the spreadsheet, along with the individual FGID symptoms reported (either by participants or as study outcome measures) and whether the instrument was reported as being validated (V) or modified from a validated instrument (m-V). The timeframe or sequence of symptom/s reporting and collection was also extracted, and whether the tool was a self-report tool or researcher-administered tool. 

Dietary assessment methods were categorised using the features shown in Table 1. Additionally, studies that reported on FGID symptom responses to a dietary protocol were included if the dietary intake measures, the dietary regime and the associated symptom-reporting methods were described. If the dietary assessment method was reported as not validated, validated or modified from a validated, this information was extracted.

A method was considered to be validated if the paper stated that a validated dietary assessment method had been use in it’s original format with a comparable study population. If validation was not mentioned, this was recorded as not reported (NR). The timeframe/sequence of dietary data collection was also extracted.

The reported relationship between dietary intake and FGID symptoms was categorised by the effect type, based on the analysis and study type. Categories were: “group by time” effect for significant differences in symptom responses to food between the control and intervention group (or between intervention arms) over the intervention period; “group” effect for significant difference in symptom responses to food between groups cross-sectionally or for retrospectively collected data; “time” effect for a significant difference in a cohort over the intervention period; “not significant if the study reported no significant group or time effect”; “not measured”; and “not applicable”.

Two researchers (GD and KD) extracted data independently and compared extracted data, with inconsistencies rechecked to achieve consensus or referred to a third reviewer for adjudication (TB).

### 2.5. Critical Appraisal

Study quality appraisal was conducted to ensure that findings from respective studies could be contextualized. Articles meeting inclusion criteria were assessed for study quality, using the Academy of Nutrition and Dietetics Quality Criteria Checklist for Primary Research standardized tool [20]. This 10-point checklist includes four relevance and six validity questions to assess population bias, study blinding, the description of the intervention and assessment tool, statistical methods, and study funding. For each criterion, outcomes are rated as being absent, present, unclear or not applicable. No studies were excluded based on quality ratings. Quality assessment was conducted independently by two reviewers (KD and TB), and consensus was achieved without a third reviewer.

### 2.6. Data Analysis

Descriptive statistics were used to analyse the study characteristics and results of included studies. For the study characteristics, mean and range calculations were applied to discrete data. Counts, sums and proportions were applied to summarise and analyse categorical data, and to generate frequency tables. Tables, graphs and numerical summary tools were used to characterise the data and to identify and display patterns when comparing dietary intake capture data and symptom-reporting data. The protocol for this review was registered with the International Prospective Register of Systematic Reviews (PROSPERO, University of York Centre for Reviews and Dissemination), with registration number CRD42018105776XXX.

## 3. Results

**Figure 1:**
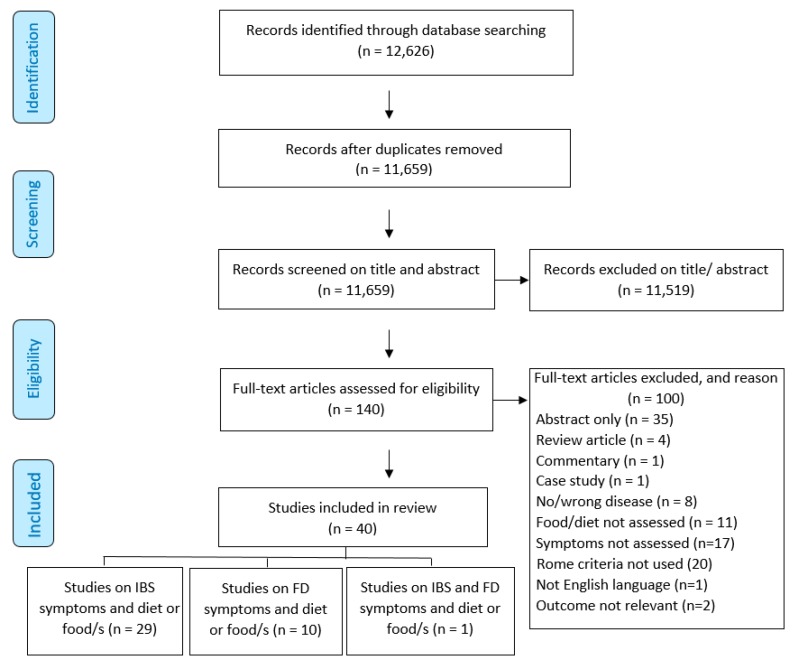
Search and screening for articles to include and exclude from a systematic review of alignment between assessment of dietary intake and functional GI disorder (FGID) symptoms.

Search strategy implementation (Supplementary Table S1) identified 12,626 citations (Figure 1). After removal of duplicates, 11,659 citations were screened on titles and abstracts. Following the screening on title and abstract, 140 articles remained for full text screening. Forty articles met the inclusion criteria and were included in this systematic review (Figure 1) [21,22,23,24,25,26,27,28,29,30,31,32,33,34,35,36,37,38,39,40,41,42,43,44,45,46,47,48,49,50,51,52,53,54,55,56,57,58,59,60]. Reasons for the exclusion of the other 100 articles are also shown in Figure 1.

**Figure 2:**
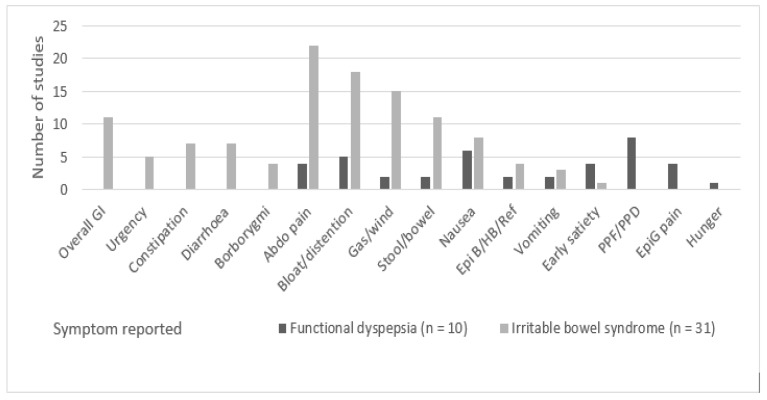
Number of included studies that reported respective IBS and FD symptoms.

**Figure 3:**
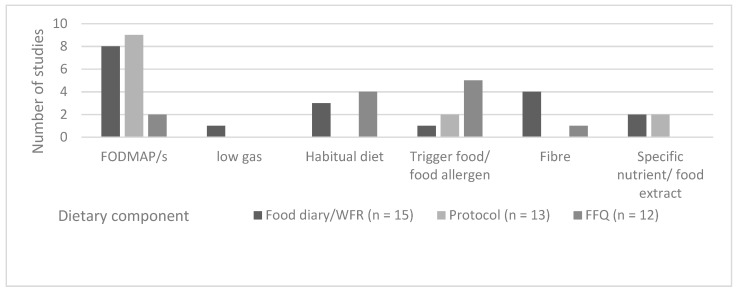
Dietary outcome measures by dietary assessment method in that assessed relationships between dietary intake and FGID symptoms.

**Figure 4:**
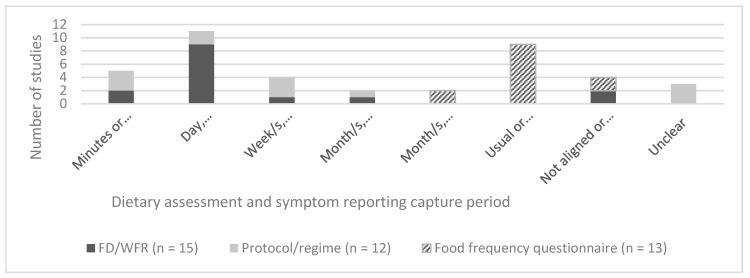
Alignment between dietary assessment and the symptom-reporting capture period in studies that assessed relationships between dietary intake and FGID symptoms.

**Figure 5:**
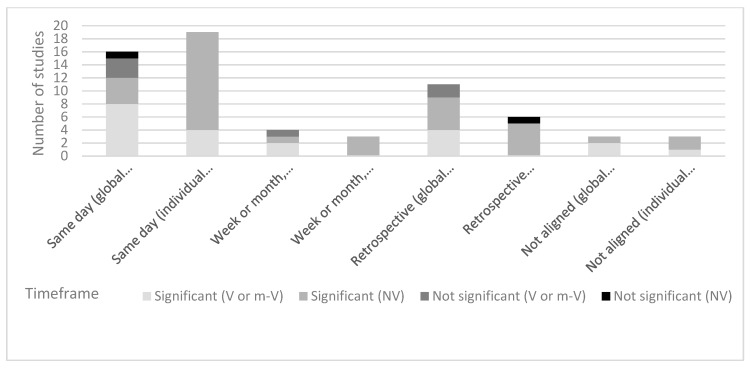
Significant and non-significant findings compared with the validation of symptom-reporting tools in studies assessing the influence of dietary intake on irritable bowel syndrome and functional dyspepsia symptoms.

The results of this review have been divided into sections. Firstly, the study characteristics, dietary assessment methods, symptom-reporting data and study quality of the 40 included studies are described in Section 3.1 and Section 3.2 and Table 2. Secondly, diagnostic criteria and symptom data are then outlined in Section 3.3, Table 3 and Figure 2. Thirdly, we examined the dietary assessment methods used in the included studies, and the results of this analysis are described in Section 3.4 and Figure 3; Figure 4. Finally, the alignment of the dietary assessment method (DAM) and symptom reporting was reviewed in Section 3.5 and Figure 5.

### 3.1. Study Characteristics

A total of 10,997 (mean 275 per study, range 20 to 4763) participants were involved in the studies, including control group participants. As shown in Table 2, eight included studies were conducted prior to 2010 [22,25,36,46,50,51,53,54], seventeen between 2010 and 2014 [23,24,27,29,31,33,38,39,41,42,43,44,45,47,55,57], and the remaining sixteen studies were conducted since 2015 [21,26,28,30,32,34,35,37,40,48,49,52,56,58,59,60]. As shown in Table 2, the 40 included studies were conducted in 20 countries—of which, 12 were conducted in Europe/United Kingdom [22,23,24,25,29,30,33,42,46,56,57,60], eleven in Scandinavia [26,27,34,35,38,39,40,41,44,47,54], six in Australasia [31,32,43,49,53,55,59], four in Asia [36,37,45,58], four in North America [28,48,50,51], and two in Iran [21,52].

### 3.2. Study Quality

Critical appraisal resulted in twenty-two included studies being rated as positive study quality [22,24,25,26,28,31,32,34,35,36,38,42,43,46,47,49,50,53,55,56,59,60] and eighteen studies rated as neutral study quality [21,23,27,29,30,33,37,39,40,41,44,45,48,51,52,54,57,58]. No studies were rated negative for study quality. Outcome measures were clearly defined and relevant in all studies, as per inclusion criteria. Of the 25 prospective studies, nine were double-blind randomised controlled trials (RCTs) [23,34,35,42,46,53,55,59,60], eight were single-blind randomised controlled trials [22,24,25,26,28,31,36,43], three were unblinded randomised controlled trials [32,47,49], two were intervention studies [50,57], one was a pre-RCT [38] and one was a case control study [56]. Of the remaining studies, thirteen were cross-sectional [27,29,30,33,37,39,40,41,44,45,51,52,54] and two were longitudinal studies [48,58]. 

### 3.3. Functional Gastrointestinal Disorder Diagnosis, Classifications and Symptom-Reporting Methods

Of the 40 included studies, 29 studies assessed diet and symptoms in IBS patients [22,23,25,26,27,31,32,33,34,35,38,39,40,41,42,43,44,45,46,47,48,49,52,53,54,55,56,59,60], including one that also investigated inflammatory bowel disease [40], one that also investigated fructose malabsorption [53], and one specific to IBS-D (Table 3) [28]. Ten studies were specific to functional dyspepsia [21,24,29,30,36,37,50,51,52,58] and the remaining study evaluated food and symptoms of both IBS and FD [57].

Rome criteria (versions I, II or III) were used in all studies to determine the patient’s diagnosis, as indicated in the study inclusion criteria and displayed in Table 3. No studies used Rome IV (2016), the most recent update of the Rome criteria. All 40 studies involved self-reporting of symptoms by participants and three [36,54,57] also involved documentation by researchers of responses to dietary challenges. The most commonly reported (or collected) symptoms for IBS were abdominal pain (*n* = 22), bloating (*n* = 18) and gas or wind (*n* = 18). Symptoms reported or collected only in IBS studies were overall GI symptoms (*n* = 11) and bowel specific symptoms including urgency, constipation, diarrhoea and borborygmi (Figure 2).

The most commonly reported or collected symptoms in FD-specific studies were postprandial fullness/distress (*n* = 8), nausea (*n* = 6) and bloating (*n* = 5). The symptoms that were reported only in FD studies were postprandial fullness/distress (*n* = 8), epigastric pain (*n* = 4) and hunger (*n* = 1) (Table 2).

The most commonly used instruments for the assessment of global symptomology were the IBS-SSS (*n* = 10 studies), validated for a capture period of the previous 10 days; the Gastrointestinal Symptom Rating Scale (GSRS) (*n* = 3), validated for capture over the previous seven days; the Birmingham IBS Symptom Score (*n* = 2 studies), validated over a capture period of the previous four weeks; and the “adequate relief” question, validated for use over the previous week (*n* = 4). The only individual symptom assessment instrument reported as being validated was the validated Bristol Stool Form (BSF), which was used in three studies. Non-validated visual assessment scales were used in 11 studies, and other non-validated symptom rating scales were used in eight studies.

The original validated IBS-SSS was the sole symptom assessment method used in four studies [27,47,59,60] and was used in a modified format in one study [23]. The IBS-SSS was used in conjunction with visual analogue scales (VASs) for individual symptoms in another three studies [32,34,35] and with the BSF used in two studies [26,40].

The validated “adequate relief” tool was used as the sole assessment method in three studies [28,53,57], and in addition to the validated GSRS in one study [55]. The GSRS was used in conjunction with non-validated individual symptom assessment tools in two other studies [28,56]. The validated Birmingham IBS Symptom Score was the overall symptomology assessment method in two studies [41,44]. The validated patient assessment of the upper gastrointestinal symptom severity index (PAGI-SYM) was the symptom assessment tool in one study [37] and the modified Rome III criteria in one study [58]. A non-validated “overall symptoms” score was used in addition to VAS for individual symptoms in two studies [38,42]. Overall symptom rating scales that were not reported as being validated were used to assess symptoms in six studies [22,25,30,39,43,45].

Visual analogue scales (VASs) for individual symptoms were the single symptom assessment method used in five studies [31,36,49,50,51]—none of which reported the VAS as being validated. VASs were used in addition to the validated BSF (*n* = 1 study) [48]. Other individual symptom rating scales that were not reported by authors as being validated were used as the single symptom assessment method in three studies [21,46,52], and four studies assessed symptom induction in relation to food consumption using a non-validated tool [24,29,33,54].

The reporting period was highly variable between studies: with daily reporting ranging from three days [45] and 42 days [31]; weekly reporting for between three [48] and eight weeks [53]; and monthly reporting for between one [56] and 16 months [40]. Overall, validated tools were used to measure global FGID symptomology, but the availability of validated tools for assessing individual symptoms was very limited.

### 3.4. Dietary Assessment Methods (DAMs) 

Prospective dietary assessment methods included food diaries of between two and 42 days duration (*n* = 13 studies [22,24,26,28,29,31,35,38,41,43,45,46,55], and weighed food record (*n* = 2) over a period of seven days [50,51]. Retrospective dietary assessment methods included food frequency questionnaires (*n* = 12) [21,25,27,30,32,33,37,39,44,52,54,56,58]—of which, five reported some form of validation [25,30,32,44,56]. Dietary protocols or regimes were used in 12 studies [23,34,36,40,42,47,48,49,53,57,59,60]. The dietary factors assessed using the respective dietary assessment methods are shown in Figure 3 and detailed in Table 2. 

### 3.5. The Alignment of Dietary Assessment and Symptom Reporting 

The dietary assessment and symptom-reporting capture periods matched exactly in 15 out of 27 prospective studies (Figure 4). Of the studies in which alignment matched at a daily level, 10 out of 16 matched exactly [24,29,36,38,43,46,50,51,57,59], three studies included a dietary intake data collection that was longer but inclusive of symptom reporting [23,31,53] and three studies involved symptom-reporting data collection that was longer but overlapped with dietary intake data collection [28,45,55]. Of the studies in which dietary assessment and symptom reporting were aligned at an interval of weeks to months, the data collection capture period matched exactly in three studies [32,42,48] and two studies included a dietary intake data collection that was longer but overlapped with symptom reporting [35,49]. Capture periods were aligned in the nine studies that reported usual dietary intake and current symptoms [21,27,30,33,37,39,44,54,58]. The capture period for dietary assessment and symptom reporting was not aligned in four studies [22,26,52,56].

Twenty five out of the 33 studies (76%) that reported on dietary intake and global gastrointestinal symptoms reported at least one significant association. Of these, 12 (36%) used a validated symptom-reporting tool, 11 (33%) used non-validated tools and three (9%) were not capture period aligned (Table 2). Of the seven studies (24%) that reported non-significant results, six (18%) used a validated global symptom assessment tool and one (3%) used a non-validated tool. Thirty out of 31 (97%) of the studies that reported on associations between individual gastrointestinal symptoms and dietary intake reported at least one significant association. Of these, four (13%) used a validated symptom assessment tool, 23 (74%) used a non-validated symptom assessment tool, and three (10%) were not aligned. The remaining study did not use a validated tool and did not report a significant finding (Figure 5). Overall, a small proportion of studies that reported significant associations between dietary factors and symptoms used a validated tool for assessing individual or global symptoms and showed alignment between the capture periods for dietary intake and symptom reporting.

## 4. Discussion

In comparing the alignment of dietary assessment and symptom-reporting capture periods, this systematic review is a novel addition to the FGID literature. Over 80% of included studies assessed dietary factors that would be expected to induce symptoms within a timeframe from minutes to hours after consumption, but the majority of these involved dietary assessment methods suited to a longer capture period. Additionally, validated tools were routinely used to measure global IBS and FD but not available for individual FGID symptom assessment. In combination, these results highlight the need for validated symptom-reporting tools that are matched with fit-for-purpose dietary assessment methods.

Although diet is now routinely implicated in symptom induction for both IBS and FD [11,12], only 40 studies were identified that had addressed associations between dietary intake assessment and FGID symptom reporting. Collation of the study characteristics showed a high representation from the United Kingdom, Scandinavia and Australasia, and a higher representation of IBS-focused studies (75%) rather than FD-specific studies (25%). Both features are consistent with broader FGID literature [61]. The high proportion of intervention studies (62.5%) compared to retrospective or cross-sectional study designs contributed to the positive study ratings for 55% of included studies. 

The use of Rome criteria for the diagnosis of the FGID was an inclusion criterion, and all 40 studies employed the most current version at the time of study implementation. The recent release of the Rome IV criteria is the likely reason that no studies reported using these diagnostic criteria [62]. There was some overlap between symptoms that were reported on in FD-specific and IBS-specific studies. As expected, the symptoms reported only in IBS-specific studies were bowel related and the symptoms only reported in FD-specific studies were epigastric pain, postprandial fullness/distress and hunger. The symptoms that were reported on in both FD-specific and IBS-specific studies were consistent with diagnostic criteria or the overlapping symptom and diagnostic profiles for these two FGIDs [62]. 

Validated instruments were used to assess global IBS and FD symptomology over capture periods ranging from one to four weeks in less than half of the included studies, and the only individual symptom-reporting tool reported as being validated was the Bristol Stool Form, used in 8% of studies. To date, individual symptom-rating scales are not validated in the same manner as the overall symptom-reporting instruments [20]. However, the high proportion of included studies (n = 19, 48%) that measured individual symptoms on a meal-by-meal or daily basis shows that such tools are more closely aligned to dietary intake capture periods for prospectively collected dietary intake data. This finding highlights the need for validated individual FGID symptom-reporting tools and for a closer consideration of symptom-reporting and dietary intake capture period alignment in future food-related FGID studies.

As expected, food diaries and weighed food records were more common in studies that investigated symptom induction or change in symptoms prospectively over time in relation to a dietary intervention. Food frequency questionnaires were generally used in studies that retrospectively investigated presence of symptoms over previous weeks or months. Food frequency questionnaires are considered suitable for assessing changes in dietary intake over time, and for ranking consumption between individuals, but lack the sensitivity to be used in studies focused on symptom induction [18]. The included dietary protocol studies that compared a specific regime to a usual care control group (or over time) were either conducted in highly controlled laboratory settings, and investigated a limited number of dietary factors, or were implemented in a free-living setting with regular dietetic support. The dietary assessment methods in these studies were less well described, with protocol adherence being the only dietary assessment measure in seven of the 12 studies, and four studies not reporting on protocol adherence in the findings.

The small proportion (17%) of studies that assessed habitual diet were suited to the use of the FFQ dietary assessment method. As food chemical intolerance symptom induction can take several weeks to become evident, FFQs may also be useful in studies investigating this dietary factor. The remaining studies assessed dietary factors that would be expected to induce symptoms within a much shorter timeframe (minutes to hours) after consumption. For example, FODMAPs [12] and fibre [11] induce symptoms from within hours up to several days and allergens have an immediate effect. However, the majority of these studies employed dietary assessment methods suited to a longer capture period, which brings the clinical significance of findings into question.

An important additional consideration in reviewing dietary assessment tools used in functional gastrointestinal disorder research is the limitations of the food composition database linked to the dietary assessment tool. A common limitation of these country-specific food composition databases is the absence or lack of specific information on fibre (e.g., resistant starches) and fermentable carbohydrates (e.g., oligosaccharides) [63]. Gaps in food composition data availability result in incomplete dietary intake information, which in turn affects the completeness and quality of dietary intake data used in FGID research. When considered in combination with the limited use of validated individual symptom-reporting tools, these findings highlight the need for improvements in the methodology of diet-focused functional gastrointestinal research.

Although the capture periods for food and symptom reporting matched or overlapped in 90% (n = 36) of studies, only 30% of studies that reported on global gastrointestinal symptoms and 13% that reported on individual gastrointestinal symptoms that identified an association with food ingestion used a validated symptom tool. Therefore, guidelines to ensure validated symptom-reporting tools are matched with fit-for-purpose dietary assessment methods are needed to minimise discrepancies in the alignment of food and symptom tools in order to progress functional gastrointestinal disorder research. 

Recommendations that would be addressed include:

(i) An explicit explanation of hypothesised relationship between food and FGID symptoms;
(ii) A clear differentiation between assessment of global symptomology and presence or induction of individual symptom;
(iii) The selection of symptom-reporting tools be based on whether the study is investigating symptom induction versus presence (or retrospective assessment) of symptoms;
(iv) The use of symptom-reporting tools that have been validated for use over the timeframe they were implemented (wherever possible);
(v) The selection of appropriate dietary assessment methodologies and the implementation of these methods by researchers with dietary assessment expertise;
(vi) The explicit explanation of the rationale for selected dietary assessment methodology, including the hypothesised association with symptom induction and the food composition database (and included nutrients) used for nutrient analysis;
(vii) For dietary protocol implementation studies, more detailed reporting of dietary intake assessment than reporting of adherence alone;
(viii) Protocols for the assessment of symptom induction in free-living settings, including dietary assessment methods that have a low participant and research burden but high specificity and sensitivity;
(ix) The reporting of both significant and non-significant findings to reduce reporting bias.

### Conclusions

This review summarises the body of research from studies aimed at assessing food and symptom relationships in IBS or FD. The findings indicate that the dietary assessment tools and symptom-reporting instruments used in these studies are often mismatched. The recommendations produced from this review are aimed at ensuring validated symptom-reporting tools are matched with fit-for-purpose dietary assessment methods to minimise discrepancies in the alignment of food and symptom tools. The implementation of these recommendations in future research will improve the determination of relationships between food ingestion and the presence or induction of FGID symptoms.

## 5. Limitations

This study is the first to report on the alignment of symptom reporting and dietary intake assessment methods in FGID research, addressed through studies that focused on the two most common FGIDs, IBS and FD. The findings will facilitate a more structured integration of dietary assessment into FGID research. Limitations associated with the review process are related to an inadequate description of methods in included studies and the need to subsequently extract and categorise this incomplete data. Examples of such limitations include the inadequate description of dietary assessment methods in dietary protocol studies and the reporting of only significant results rather than both significant and non-significant results, particularly in intervention studies with a control group. Recommendations to improve or reduce the impact of these limitations have been outlined in this review. It is also acknowledged that the wide range and large number of dietary factors and symptoms reported within studies increased the likelihood of at least one significant association being identified. Another consideration in FGID research is that study participants are likely to have made dietary changes prior to study participation, which may affect the assessment of usual dietary intake. For example, the reported intake in an FFQ (over previous three months) may not be sensitive to foods that have been reduced or eliminated by those with FGIDs prior to that dietary assessment capture period. It is important to take these factors into consideration when assessing study findings, along with the reported misalignment of dietary assessment and symptom-reporting instruments.

## Figures and Tables

**Table 1:** Characteristics and defining features used for categorisation of dietary assessment methods.

Dietary Assessment Method	Recall Period and Method	Recorded by	Usual Location	Usual Collection Period	Type of Validation
Food diary	Prospective: Actual intake collected per meal or day	Self-reported by participant	Free living	Between 1 day and up to several weeks	No
Weighed food record	Prospective: Actual intake collected per food item	Self-reported by participant or researcher report	Free living or lab	Between 1 meal and 3 days	Criterion or relative
24 h recall	Retrospective: Recall previous 24 h	Interviewer administered or self-reported	Free living	24 h (often 3 non-consecutive days)	Relative
Food history	Retrospective: Recall of usual intake	Often dietitian-administered	Lab or clinic	Previous week or usual week	No
Food frequency questionnaire	Retrospective: Usual frequency of 30–150 foods listed	Self-reported	Free living or lab	Previous 3–6 months	Relative
Food questionnaire	Retrospective: Questions about frequency of food items	Self-reported	Free living or lab	Usual intake	No

**Table 2:** Study characteristics, dietary assessment methods and measures, and symptoms assessment tools and measures for a scoping review of diet and symptom capture periods.

Author (Year) Country	Sample Size Study Design Study Quality	FGID Diagnostic Criteria	Dietary Factor	Dietary Assessment Method (DAM) and Measures	DAM Capture Period	Symptom-Reporting Method and Measures	Symptom Recording Period	Timeframe Alignment	Significant Association
**Studies that used weighed food record DAM**									
Pilichie-wicz (2009) USA	*n* = 41 Cross-sectional Quality (+)	FD Rome II	Dietary intake/dietary patterns/nutrients	DAM: A 7 day WFR Measures: Energy intake, amount eaten, macronutrients (g), distribution of fat/carbohydrate/protein/ alcohol (%) Self-report	In total, 7 days	Symptoms: Abdominal pain, cramps, bloating, nausea, postprandial fullness Measures: SSS (1–10), from not influencing (1–3); modest (score, 4–7), diverting from; or strong (score, 8–10), impairing activities of daily living reported within and after 2 h post meal	For 7 days	Hours, prospective	Y
Pilichie-wicz (2008) USA	*n* = 16 Non-randomised intervention Quality (+)	FD Rome II	High-fat (high-FAT) meal vs. high-carbohydrate (high-CHO) meal (500 kcal/400 g each), or a low-nutrient control meal (180 kcal/400 g)	DAM: A WFR over test period Measures: Energy kilocalories, carbohydrate, and protein (% energy, grams)	In total, 7 days of intervention period	Symptoms: Nausea, bloating, abdominal discomfort, pain, fullness, and hunger Measures: 100 mm VAS score for five symptoms at 0, <60 and 60–90 min post meal	Baseline, and before, 60 min and 90 min after each of the three test meals.	Hours, prospective	Y
**Studies that used food diary DAM**									
Eswaran (2016) USA	*n* = 92 Single-centre RCT Quality (+)	IBS-D Rome III	Low FODMAP diet vs. modified National Institute for Health and Care Excellence guidelines (mNICE)	Prospective 3 day food diary (and one 24 h dietary recall) Measures: Energy (kilocalories), FODMAPs, macronutrients, fibre, and alcohol (grams) Self-report	In total, 0, 2 and 4 weeks	Symptoms: Abdominal pain, bloating, urgency, stool consistency and frequency Measures: “Adequate relief of overall IBS symptoms” assessed weekly. Daily abdominal pain score (a ≥30% reduction). Daily BSF value of ≥1 compared with baseline for 2/4 weeks). Changes from baseline in abdominal pain score, bloating score, urgency score, stool consistency as measured by BSF, and stool frequency, averaged over each treatment week. Self-report	Daily symptom assessment, adequate relief assessed weekly	Daily, prospective (diet subset of SR)	Y
Laatikain-en (2016) Finland	*n* = 87 Randomised double-blind controlled crossover study Quality (+)	IBS Rome III	Low-FODMAP rye bread vs. regular rye bread	DAM: 4 day food records for run-in and treatment periods. Adherence via tick-box diary during treatment periods and from food records. Measures:Energy (kilocalories) macronutrients, fibre, starch, sodium (grams) Self-report	Baseline and 2 x 4 day out of 10 day trial each study period	Symptoms: 10 IBS symptoms Measures: IBS-SSS (total) and 10 symptoms were recorded (VAS 0–100 mm for each) at the baseline (week 0) and during study weeks 1, 2, 3 and 4 during both study periods. A total symptom score (= mean of all ten symptoms) calculated weekly. Mean values of weeks 1, 2, 3 and 4 calculated for individual symptoms (flatulence, abdominal pain, cramps, stomach rumbling) and for total symptom score. Not validated but cited as previously used. Self-report	IBS-SSS at 0, 2 and 4 weeks (past 10 days). Self-report	Weekly, prospective (SR subset of DAM at daily level)	Y
Böhn (2015) Sweden	*n* = 75 Parallel, single-blind RCT Quality (+)	IBS Rome III	Low FODMAPs (n = 38) or “IBS diet” (n = 38) (regular meals, avoid large meals, reduced fat, low insoluble fibres, low caffeine, and low gas-producing foods)	A 2 × 4 day food diary Measures: Energy, macronutrients, fibre, monosaccharides, FODMAPs, and pectin Self-report	In total, 4 days baseline and 4 days post study (4 weeks)	Symptoms: Stool formation, IBS symptoms Measures: BSF, IBS-SSS >175 (moderate to severe symptoms) at baseline for inclusion Self-report	BSFS (daily), IBS-SSS (day 0, 14, and 28).	Weekly, Prospective BSF (daily)	Y
Pilichie-wicz (2009) USA	*n* = 41 Cross-sectional Quality (+)	FD Rome II	Dietary intake/dietary patterns/nutrients	DAM: A 7 day WFR Measures: Energy intake, amount eaten, macronutrients (g), distribution of fat /carbohydrate/protein/alcohol (%) Self-report	In total, 7 days	Symptoms: Abdominal pain, cramps, bloating, nausea, postprandial fullness Measures: SSS (1–10), from not influencing (1–3); modest (score, 4–7), diverting from; or strong (score, 8–10), impairing activities of daily living reported within and after 2 h post meal	For 7 days	Hours, prospective	Y
Pilichie-wicz (2008) USA	*n* = 16 Non-randomised intervention Quality (+)	FD Rome II	High-fat (high-FAT) meal vs. high-carbohydrate (high-CHO) meal (500 kcal/400 g each), or a low-nutrient control meal (180 kcal/400 g)	DAM: A WFR over test period Measures: Energy kilocalories, carbohydrate, and protein (% energy, grams)	In total, 7 days of intervention period	Symptoms: Nausea, bloating, abdominal discomfort, pain, fullness, and hunger. Measures: 100 mm VAS score for five symptoms at 0, < 60 and 60–90 min post meal	Baseline then before, and 60 min and 90 min post each of the three test meals.	Hours, prospective	Y
Azpiroz (2014) Spain	*n* = 30 Single-blind RCT Quality (+)	PDS, IBS Rome III	Low-flatulogenic vs. Mediterranean diet	DAM: Daily food diary Measures: Calories (kcal) fibre (grams) Self-report	In total, 7 days	Symptoms: Daily anal gas evacuations and severity Measures: Severity of gas-related symptoms (0–10), Digestive comfort scale -5 (unpleasant) to +5 (pleasant) Self-report	Daily for 10 days (3 days prior to and or 7 days of trial)	Daily, prospective	Y
Halmos (2014) Australia	*n* = 38 Single-blind, crossover RCT Quality (+)	IBS Rome III	Less than 0.5 g intake of FODMAPs per meal (low FODMAPs) vs. typical Australian diet	DAM: Food diary Energy (Megajoules), macronutrients, sugar, starch, fibre, and FODMAPs (grams) Self-report	In total, 2 x 21 day food diary with 21 day washout	Symptoms: Overall gastrointestinal symptoms, abdominal pain, bloating, passage of wind, and dissatisfaction with stool consistency Measures: Daily symptoms rated using a 0 to 100 mm VAS score. Differences of 10 mm or more considered clinically significant. Self-report	Overall and individual symptom rating daily in weeks 2 and 3 each intervention	Daily, prospective (SR subset of DAM at daily level)	Y
Mazzawi (2013) Norway	*n* = 46 Retrospective cross-sectional Quality (N)	IBS Rome III	Usual diet vs. diet after dietary guidance (Low FODMAP and fibre)	DAM: Food diary (2 weeks usual intake pre-trial) and FFQ (226 item) Measures: Energy (kilojoules), macronutrient (% energy), fibre (grams) and micronutrients	Past 3 months	Symptoms: Pain (three items), diarrhoea (five items) and constipation (three items) Measures: Birmingham IBS symptom score: (11 IBS symptom questions: 6-point Likert scale, 0 (never) to 5 (all of the time); lower scores = fewer symptoms. Self-report	Past three months	Months (3), retrospective	Y
Staud-acher (2012) Australia	*n* = 41 RCT double-blinded, four-arm randomized trial Quality (+)	IBS Rome III	Four weeks of fermentable carbohydrate restriction	DAM: A 7 day food diary Measures: Total energy (Kcal), macro-nutrients (g), starch (g), micronutrients (mg), and FODMAPs (g)	Week 0 and Week 4	Symptoms: bloating, abdominal pain, flatulence, borborygmi, urgency, diarrhoea, constipation, incomplete evacuation, heartburn, nausea, and lethargy Measures: Gastrointestinal symptom rating scale of each day using a 4-point scale (absent, mild, moderate, severe). Adequate relief question weekly	GSRS for 7 days, adequate relief question on 7th day (Week 0 and 4)	Daily, prospective	Y
Filipovic (2011) Serbia	*n* = 180 Cross-sectional Quality (N)	FD Rome II	All food eaten, drinks consumed, plus a standardized questionnaire to identify eating habits and dietary patterns reported to exacerbate or relieve GI symptoms	DAM: A 7 day diet diary Measures: Food item consumption Self-report	In total, 7 days	Symptoms: Epigastric pain and burning, heartburn, postprandial fullness, bloating and early satiety Measures: Occurrence of specific dyspeptic symptoms reported to be exacerbated or relieved by food/eating. Self-report	Daily for 7 days	Daily, prospective	Y
Ligaarden (2011) Norway	*n* = 17 Run-in to RCT Quality (+)	IBS Rome II	Dietary intake for macro- and micronutrients	DAM: A 7 day food diary Measures: Energy (kilojoules), macronutrient (% energy), fibre (grams) and micronutrients Self-report	In total, 7 days	Symptoms: Abdominal pain/discomfort, urgency, and bloating Measures: Symptoms graded as none, mild, moderate, or severe (scores 0–3), recorded on diary cards in the evening for 7 days. IBS sum score calculated (0–15). Self-report	Assessed daily	Daily, prospective	Y (Vitamin B6 only)
Ong (2010) Australia	*n* = 30 Randomised, single-blind, crossover trial Quality (+)	IBS Rome III	Low (9 g/day) or high (50 g/day) FODMAPs diet for 2 days each	DAM: Food diary Measures: Total energy (kJ), macronutrients (grams), individual FODMAP (grams), fibre (grams) and resistant starch (grams) Self-report	In total, 7 days run-in; 2 days (A); 7 days wash out, 2 days (B)	Symptoms: Abdominal pain, bloating and wind Measures: Composite symptom score, adding 3-point Likert scale ratings (0–3): 0 = none, 1 = mild, 2 = moderate and 3 = severe	Daily for 18 days	Daily, prospective	Y
Park (2010) South Korea	*n* = 95 Secondary analysis, cross-sectional Quality (N)	IBS Rome I	Usual intake	DAM: Food record: time of day, quantity, and type of food and beverage consumed Measures: Macronutrients, Micronutrients, sugar type, alcohol, and caffeine Self-report	In total, 3 days: Mid luteal menstrual phase	Symptoms: bloating (minimal, mild, moderate) abdominal pain, intestinal gas, constipation, diarrhoea, heartburn, indigestion, nausea, and stomach pain. Measures: Daily symptoms rating as: not present (0), minimal (1), mild (2), moderate (3), and extreme (4). Self-report	One full menstrual cycle plus 5 days	Daily, prospective (diet 3 day subset of SR one month)	Y –fructose only
Aller (2004) Spain	*n* = 56 Single-blind RCT Quality (+)	IBS Rome II	Group 1: 10.4 g/d of fibre/day; Group 2: 30.5 g/d of fibre/day	DAM: A 3 day written food diary Measures: grams of fibre Self-report	In total, 3 days	Symptoms: Self-reported abdominal pain (frequency and severity), bowel score (defecation frequency, defecation straining, incomplete evacuation, and laxatives), and general symptoms (nausea, vomiting, flatus, and bloating) Measures: 0–5 Likert scale Assessment period NR. Self-report	Baseline and 3 months (not specific to food diary collection)	No	No – improved both groups
Parker (2001) UK	*n* = 122 Double-blind, placebo-controlled challenges Quality (+)	IBS Rome criteria (NR)	Subgroups: Low lactose diet (positive breath test + placebo trial) or exclusion diet or low fibre for non- responders For breath teas negative: exclusion diet then reintroduction of foods (testing each over 2 days) for possible intolerances Low fibre diet alternative to elimination: 11 g non-starch polysaccharide	DAM: Food and symptom diary Measures: Dietary adherence vs. symptoms Self-report	In total, 3 weeks	Symptoms: Abdominal pain, number of bowel motions daily, urgency to defecate, consistency of faeces, flatulence, headache, abdominal distension, well-being. Measures eight symptoms scored 0 to 4 (0 = no symptoms and 4 = most severe, urgency scored from 0 to 3). Self-report	For 3 weeks (daily)	Daily, prospective	N
**Studies that used questionnaire (including FFQ) DAM**									
Harvie (2017) New Zealand	*n* = 50 Randomised unblinded, delayed control intervention trial Quality (+)	IBS Rome III	Low FODMAP diet, followed by the systematic reintroduction of individual FODMAPs	DAM: FODMAP-specific FFQ at 0, 3 and 6 months Measures: Total FODMAP sum of fructo-oligosaccharides, galacto-oligosaccharides, lactose, excess fructose to glucose, sorbitol, and mannitol	In total, 6 months	Symptoms: Bloating and abdominal pain IBS-SSS Scores range from 0–500, subscales for bloating and severity (0–100) and frequency of abdominal pain (days in 10: 0–10). Total scores and individual symptoms scores used in analysis Self-report	For 0, 3 and 6 months	Monthly, retrospective	Y
Tigchelaar (2017) Netherlands	*n* = 380 Case control Quality (+)	IBS Rome (NR)	Habitual diet of IBS vs. control participants	DAM: A 148-item FFQ (linked to Dutch Food Composition Database) Measures: Fruit, vegetables, fibre, docosahexaenoic acid, eicosapentaenoic acid, saturated fatty acids, trans fatty acids, sodium and alcohol in grams	Past 4 weeks	Symptoms: Abdominal pain, reflux, diarrhoea, constipation, and indigestion Measures: GI symptoms (14 day end-of-day diary, 1–5 point scale) and Gastrointestinal Symptom Rating Scale (1–7 point scale) for abdominal discomfort, abdominal pain, constipation, diarrhoea, bloating, flatulence, belching and nausea. GSRS total score = sum of all syndromes.	For 14 days	General, retrospective (diet) prospective (symptoms)	Y
Xu (2017) China	*n* = 1139 Longitudinal Quality (N)	FD mROME III (Chinese)	Dietary behaviours in the previous two months	DAM: Researcher-administered FFQ (specific food frequency and patterns) Measures: a) diet patterns (> 3 times per week): irregular mealtime, skipping breakfast, night snacking, and dining out; b) food frequency 3+ times/week): raw food or unboiled water, fatty food, spicy food, regular coffee or tea regularly, and alcohol intake Self-report	Past two months	Symptoms: Postprandial fullness, early satiation, upper abdominal pain, upper abdominal burning, reflux and nausea. Measures: mROME III only	Past 3 months (Rome III)	General, retrospective	Y
Göktaş (2016) Turkey	*n* = 303 Comparative cross-sectional Quality (N)	FD Rome III	Nutritional intake and habits	DAM: A 34 item FFQ (foods expected to exacerbate FD symptoms) Measures: Frequency of intake per item Self-report	Current intake	Symptoms: Epigastric pain symptom at least moderate severity, postprandial distress syndrome symptom (distress after a regular sized meal or early satiety) or a mix of both conditions Self-report	Survey of symptom induction by food in FFQ	General, retrospective	NA
Lee (2016) Korea	n = 121 Cross-sectional Quality (+)	FD Rome III	Spicy food intake: green chili, red pepper paste, Kimchi, rice cakes in hot sauce, and other food containing chilli or red pepper	DAM: Spicy food intake score (NV) Measures: Scores (0 to 5) almost none = 0, a few times/month = 1, few times/week = 2, 1/day = 3, 2/day = 4, and ≥ 3/day = 5. Self-report	Usual intake	Symptoms: Stomach fullness, abdominal distention, and retching. Measures: Assessment of gastrointestinal symptoms (PAGI-SYM), severity recorded as 0 (none), 1 (very mild), 2 (mild), 3 (moderate), 4 (severe), and 5 (very severe) Self-report	Cross-sectional Self-report	General, retrospective	Y
Saneei (2016) Iran	*n* = 4763 Repeat cross-sectional Quality (N)	CUD Rome III (Persia)	Spicy food consumption and chronic uninvestigated dyspepsia (CUD)	DAM: A self-administered questionnaire Measures: Frequency and regularity of meals, drinking fluids with meals, and weekly intake of spicy food (curry, ginger, cinnamon, chili pepper, turmeric, etc.) Options: “never”, “1–3 times”, “4–6 times”, “7–9 times” or “> 10 times” Self-report	Baseline (usual intake)	Symptoms: Distressing postprandial fullness, early satiation and/or epigastric pain or epigastric burning Measures: Four-item rating scale (i.e., “never” or “rarely”, “sometimes”, “often”, and “always”)	One month (no overlap with diet questionnaire)	N	Y
Akhondi-Meybodi (2015) Iran	*n* = 384 Cross-sectional Quality (+)	FD Rome III	“Aggravating” and “alleviating” foods	DAM: Questionnaire specific to study (NV) Measures: 114 commonly eaten foods Self-report	Usual intake	Symptoms: Pain, defecation, nausea, and vomiting Measures: A 4-point scale for either “aggravation” OR “alleviation,” rated as: low, medium, high, and very high (NV) Self-report	Retrospective— “Associated with food ingestion”	General, retrospective	NM
Hayes (2014) Ireland	*n* = 246 Cross-sectional Quality (N)	IBS Rome III	Self-reported food intolerance. Differentiated between specific foods and eating, and time to symptom induction after eating	DAM: Tailored FFQ questionnaire (NV) Measures: Specific foods and time between eating and symptoms Self-report	Current intake	Symptoms: Pain, bloating, distension, diarrhoea, constipation, and to specify the time between food intake and symptom onset. Measures: Select food-induced symptoms from a list Self-report	Cross-sectional–retrospective, timeframe NR	General, retrospective	NA
Böhn (2013) Sweden	*n* = 197 Cross-sectional Quality (+)	IBS Rome III	Foods containing amines; birch-related; benzoic acid; capsaicin; grass-related; histamine-releasing; latex-related; sulphites, fermentable carbohydrates; lectins; mugwort-related; mite-related; ragweed	FFQ: 56 food items (NV) Measures: average daily intakes for energy, macronutrients, and selected micronutrients (grams and % energy) Self-report	Usual (NR)	IBS symptoms (IBS Severity Scoring System) Self-report	Retrospective— “Associated with food ingestion”	General, retrospective	Y
Wilder-Smith (2013) Switzerland	*n* = 312 Intervention Quality (N)	FGIDs Rome III	Four-week dietary adaptation, 1 week of low saccharides and polyols, weekly introduction of specific foods containing fructose, fructan, inulin and lactose to determine individual tolerance. Lactose and fructose challenges 4 days apart	DAM: (1) Questionnaire and interview: (2) Maintain below threshold for breath testing Measures: Adequate compliance = adhered to diet for at least 50% of the meals consumed Self-report and researcher-reported	In total, 4 weeks: threshold In total, 1 week: breath tests	Symptoms: FGID (abdominal distension, flatulence, fullness, nausea, diarrhoea, abdominal cramps, borborygmi) Measures: Average relief 10-point Likert scales and proportion reporting “Adequate global symptom relief” and average relief on the 10-point symptom scale. Intolerance > 2 over baseline using a symptom score index, which was scored hourly concurrently with the collection of the breath samples (0–5 h)	For 4 weeks: threshold 1 week: breath tests	Hourly (breath tests) prospective	Y
Ligaarden (2012) Norway	*n* = 388 Cross-sectional Quality (+)	IBS Rome II	Beverages (milk, water, carbonated beverages, and alcoholic beverages), fruits, vegetables, fatty fish, cheese, and omega-3 fatty acid supplements	DAM: FFQ—usual intake Measures: Frequency Self-report	Usual intake	Severity of symptoms (score 1–12) calculated as the product of severity (mild, moderate, severe (score 1–3)) and frequency (one day or less per week, two to three days per week, four to five days per week, more than five days per week (score 1–4)) Self-report	Cross-sectional-current symptoms	General, retrospective-	Y
Ostgaard (2012) Norway	*n* = 114 Retrospective, cross-sectional Quality (+)	IBS Rome III	Usual intake after dietary guidance for low FODMAP vs. no guidance	DAM: FFQ Measures: Macronutrients, micronutrients, and FODMAPs Self-report	Past 2 years	Symptoms: Pain (three items), diarrhoea (five items) and constipation (three items) Measures: Birmingham IBS symptom score: (11 IBS symptom questions: 6-point Likert scale, 0 (never) to 5 (all of the time), and lower scores = fewer symptoms. Self-report	Past 2 years	Years (2), retrospective	N
Bijkerk (2009) Netherlands	*n* = 275 Single-blind RCT Quality (+)	IBS Rome II	Twelve weeks of treatment with 10 g psyllium, 10 g bran, or 10 g placebo (rice flour)	DAM: A 78 item FFQ for fibre, validated for ranking participants Adherence checked Self-report	In total, 12 weeks assessed monthly	Symptoms: Validated “adequate relief” of IBS-related abdominal pain or discomfort Measures: Validated symptom score (VAS for five aspects of bowel dysfunction and IBS symptom intensity. Self-report	Monthly at 4, 8, 12 weeks “adequate relief 2 out of 4 weeks”	Monthly, retrospective	Y
Simrén (2001) Sweden	*n* = 410 Cross-sectional Quality (N)	IBS Rome I	GI symptom “trigger” food intake	DAM: FFQ Measures: 35 reported trigger foods Self-report	Current intake	Symptoms: GI symptoms related to 35 meals and individual foods were described by participants (then grouped). Measures: Mean time from food intake until symptoms appeared and severity of subjective GI symptoms assessed using a 5-step scale ranging from no (0), mild (1), moderate (2), severe (3) to very severe (4) symptoms.	Associated with food (cross-sectional)	General, retrospective	Y
**Studies that used a dietary protocol**									
Hustoft (2017) Norway	*n* = 20 Randomized, double-blinded, placebo-controlled, Crossover trial Quality (+)	IBS Rome III	Nine-week low FODMAP diet with 3 week run-in, then 10 days of either (A) 16 g/d of fructo-oligosaccharide (FODMAP), a 3 week washout, then (B) 16 g/d of maltodextrin (placebo) or reverse sequence	DAM: Dietary protocol (Baseline, after 3 weeks of low FODMAP, after 10 days with (A/B), after a 3 week washout period, after 10 last days with (A/B)) Measures: By A/B vs. symptoms Self-report	In total, 9 weeks (compliance not reported)	Symptoms: Severity of abdominal pain, frequency of abdominal pain, severity of abdominal distension, dissatisfaction with bowel habits, and interference with daily life. Measures: IBS-SSS: five items on a 100-point VAS; overall score (0–500) to classify severity. ↓ 50 points = significant treatment response. Reporting of nausea and/or vomiting, early satiety, headache, backache, tiredness, belching and/or passing gas, heartburn, frequent or sudden urge to urinate, thigh pain, and muscle/ joint pain using a 100- point VAS. Self-report	IBS-SSS and symptoms previous 10 days. Global question symptom relief satisfaction past 7 days.	Weekly, prospective SR vs. diet protocol (SR subset of diet compliance)	Y
Maagaard (2016) Denmark	*n* = 131 (IBS) Retrospective cross-sectional Quality (+)	IBS and IBD Rome III	Low FODMAP diet	DAM: FODMAP adherence reporting scale: five questions, response options (never, rare, sometimes, often, always; each 1—5) = maximum score of 25 points. Measures: Score of at least 20 points (≥ 80%) = adherent to the diet. In addition, FODMAP diet satisfaction questionnaire Self-report	Past 16 months	Symptoms: Abdominal pain, bloating, constipation, diarrhoea, borborygmi, nausea/vomiting, and fatigue. Measures: Validated IBS-SSS using VAS-100 Self-report	Past 16 months	Months (16), retrospective	Y
Peters (2016) Australia [49]	*n* = 74 Randomised controlled trial Quality (+)	IBS Rome III	Comparison of hypnotherapy to the low FODMAP diet on GI symptoms	DAM: Dietary protocol Measures: Adherence = up to three accidental exposures to high FODMAP foods in 6 week study period. Self-report	In total, 6 weeks	Symptoms: Abdominal pain, bloating, wind, stool consistency and nausea. Measures: IBS-SSS, VAS 0–100 after 6 weeks and 6 months. Self-report	After 6 weeks and 6 months	Months, retrospective	Y
Portincasa (2016) Italy	*n* = 121 Randomized, double-blind, placebo-controlled trial Quality (+)	IBS Rome III	Dietary supplementation with Curcumin (84 mg) and Fennel seed (50 mg) oil daily for 30 days	DAM: Dietary supplement protocol only Measures: Compliance with supplementation protocol, and adverse events Self-report	In total, 30 days	Symptoms: Presence and intensity of abdominal pain and bloating, relief following defecation, and impact on QOL, and days of symptoms preceding 10 days. Measures: IBS-SSS five items on a 100-point VAS for severity (total score 0–500). IBS-SSS reduction level of 50 points was considered as an improvement. Self-report	For 30 days	Daily, prospective	Y
Yao (2015) Australia	*n* = 41 Double-blinded placebo control RCT Quality (+)	IBS Rome III	In total, 10 g sorbitol, mannitol or glucose ingestion	DAM: Study protocol—researcher administered Measures: Compliance Researcher-reported	In total, 4 h each test	Symptoms: Overall gastrointestinal symptoms, abdominal pain/discomfort, bloating and wind. Measures: IBS-SSS, 100 mm VAS visual analogue scale of severity from 0 (no symptoms) to 100 mm (worst it has been). Composite score calculated from individual symptom scores, corrected for baseline symptoms.	Previous 9 days (baseline) then pre- and 4 h post-ingestion for	Hourly, prospective	Y
Pedersen (2014) Denmark	*n* = 123 Randomised, unblinded controlled trial Quality (+)	IBS Rome III	Low FODMAP diet vs. Lactobacillus Rhamnosus vs. Danish/Western diet	DAM: Dietary protocol Measures: Adherence not reported Dietitian support during trial Self-report	In total, 6 weeks	Symptoms: Abdominal pain, frequency of abdominal pain, severity of abdominal distension, dissatisfaction with bowel habits, and interference with life in general. Measures: IBS-SSS five items on a 100-point VAS for severity (total score 0–500). IBS-SSS reduction level of 50 points was considered as an improvement. Self-report	Weekly	Weekly, prospective	Y
Pérez y López (2015) Mexico	*n* = 31 Longitudinal Quality (N)	IBS Rome III	Low FODMAP diet by IBS subtype	DAM: Dietary protocol Measures: Written meal plan adherence Self-report	Past 3 weeks	Symptoms: Abdominal pain, bloating, and flatulence (VAS) Measures: Graded for severity on a scale from 0 to 10. Global satisfaction scale: 5-point scale. BSF results quantified. Self-report	Weekly for 3 weeks	Weekly, prospective	Y
Aydinlar (2013) Turkey	n = 21 Double-blind, cross-over RCT Quality (N)	IBS Rome III	IgG antibody tests against 270 food allergens. Tailored IgG provoking or elimination diet for 6 weeks each (crossover)	DAM: Diet adherence (intrusions NR) Measures: Allergen food consumption Self-report	In total, 12 (2 × 6) weeks	Symptoms: IBS symptom diary (modified from IBS-SSS) Self-report	Daily for last 10 days of 2 × 6 weeks intervention	Daily, prospective (SR subset of DAM)	Y–pain/bloating
Moritz (2013) Austria	*n* = 320 Placebo control double-blind crossover RCT Quality (+)	IBS Rome II	Lactose or fructose elimination diet	DAM: Dietary protocol Measures: Allocation to group, adherence NR Self-report	In total, 3 weeks each	Symptoms: Abdominal pain severity, number of days with abdominal pain, bloating/flatulence, and contentment Measures: VAS graded from 0 (no perception) to 100 (very strong perception) for total symptom score Self-report	Post each three-week period	Weeks (3), retrospective	Y
Shepherd (2008) Australia	*n* = 25 Double-blinded, randomized, four-arm, placebo-controlled rechallenge trial Quality (+)	IBS and fructose malabsorption responsive to low FODMAP diet) ROME II	A 22 week individually energy tailored low FODMAP diet (food provided). Test drink containing (low/medium/high) dose (g/day) of fructan, fructose, or glucose, 3/day at 50 mL/meal for 3 days (low), 100 mL for 3 days (medium), or 170 mL (high) for rest of 2 weeks test if tolerated. A 10 day washout between each of the four tests.	DAM: Diary entries (timing and volume) of ingested test drinks, numbers of used and unused bottles counted to assess adherence Measures: Total energy (KJ), macronutrients (g), micronutrients (g), and FODMAPS (g) Self-report	In total, 22 weeks including baseline, 4 × 2 week test periods and washouts	Symptoms: Overall abdominal symptoms, wind, bloating, abdominal pain, tiredness, and nausea (at highest tolerated dose) Measures: Adequate relief question + daily symptom diary (based on VAS-100 mm) each symptom	Daily to highest tolerated dose	Daily, prospective	Y
Lee (2006) Korea	*n* = 42 Randomised crossover study Quality (+)	FD Rome II	In total, 500 mL of non-nutrient water or caloric nutrient drink (1 kilocalorie/mL, carbohydrate 64%, protein 14%, and fat 22%) in randomized order	DAM: Dietary protocol of 100 mL/minute for 5 min, stopping if nausea, discomfort or pain induced Measures: Volume consumed Researcher administered	In total, 30 min	Upper abdominal symptom severity post water or nutrient drink. A 100 mm VAS from 0 defined as none; 100 as worst severity imaginable. Symptoms included fullness, bloating, nausea, belching and epigastric pain measured immediately before and at 5 min intervals after ingestion of the test meal. Self-report	Sum of scores for each symptom during 30 min postprandial period. Self-report	Minutes, prospective (5 min intervals vs. protocol)	Y

BSF: Bristol stool form, CHO: carbohydrate, FD: functional dyspepsia, FFQ: food frequency questionnaire, FGIDs: functional gastrointestinal disorders, FODMAP: fermentable oligosaccharides, disaccharides, monosaccharides and polyols, g/d: grams per day, GI: gastrointestinal, IBS: irritable bowel syndrome, IgG: immunoglobulin G, ml: millilitre, mm: millimetre, mRome: modified ROME criteria, N: Negative, NA: not applicable, NM: not measured, NR: not reported, NV = not validated, Quality (+) = Study quality rated as positive using the American Dietetic Association Evidence Analysis Manual, Quality (N) = Study quality rated as neutral using the American Dietetic Association Evidence Analysis Manual, RCT = Randomised controlled trial, SSS: symptom severity score, SR: symptom reporting, V: validated, VAS = visual analogue scale, and WFR: weighed food record.

**Table 3:** Diagnostic criteria used in studies that assessed the relationship between food and functional gastrointestinal disorders.

Diagnostic Criteria	*n*	Studies
ROME I—IBS	2	[45,54]
ROME II—IBS	6	[22,25,38,39,42,53]
ROME II—FD	4	[29,36,50,51]
ROME III—IBS	23	[23,24,26,27,28,31,32,33,34,35,40,41,43,44,46,47,48,49,55,56,57,59,60]
ROME III—FD	6	[21,24,30,37,52,57,58]

## Supplementary Materials

The following are available online at https://www.mdpi.com/2072-6643/11/11/2590/s1: Table S1: Systematic review protocol of dietary factors that influence functional dyspepsia, Table S2: Participants, interventions, comparisons, outcomes and study design (PICOS) used to systematically review relationship between food ingestion and symptom reporting in functional gastrointestinal disorders, Table S3: PRISMA checklist for systematic review of the alignment of dietary intake and symptom-reporting capture periods in studies assessing associations between food and functional gastrointestinal disorder symptoms.
