# Supplementary material for: The Alignment of Dietary Intake and Symptom-Reporting Capture Periods in Studies Assessing Associations between Food and Functional Gastrointestinal Disorder Symptoms: A Systematic Review
Source: Nutrients. 2019 Oct 28;11(11):2590. doi: 10.3390/nu11112590 (PMC6893476; doi:10.3390/nu11112590)
Supplement: Supplementary file 1 [file nutrients-11-02590-s001.zip › nutrients-613632-SI/S2 PICOS DA_SR_SR.pdf]

Supplementary Table 2: Participants, interventions, comparisons, outcomes and study design (PICOS) used to systematically review relationship between food ingestion and symptom reporting in functional gastrointestinal disorders

PICOS concepts	Criteria for systematic review
Participants	Adults (18 years and over) with diagnosed, uncomplicated irritable bowel syndrome or functional dyspepsia
Interventions	Food ingestion
Comparisons	Not applicable
Outcomes	Symptom induction or presence of symptoms
Study Design	RCT, cohort, cross sectional
