# Supplementary material for: The Alignment of Dietary Intake and Symptom-Reporting Capture Periods in Studies Assessing Associations between Food and Functional Gastrointestinal Disorder Symptoms: A Systematic Review
Source: Nutrients. 2019 Oct 28;11(11):2590. doi: 10.3390/nu11112590 (PMC6893476; doi:10.3390/nu11112590)
Supplement: Supplementary file 1 [file nutrients-11-02590-s001.zip › nutrients-613632-SI/Supplementary Table 1 Search strategy DAM and FGID.pdf]

Supplementary Table 1: *Systematic review protocol of dietary factors that influence functional dyspepsia*

Searches conducted Monday 22nd April 2019 in the following databases: MEDLINE, Medline in process, EMBASE, PsycINFO and the Cochrane central register of controlled trials.

Example Database(s): MEDLINE 2000 to Present with Daily Update

Search Strategy:

#	Searches
1	Dyspepsia/
2	Heartburn/
3	Eructation/
4	(acid reflux or heartburn or regurgitation or belch* or bloat* or indigestion).tw.
5	((disturb* or disorder* or dysfunction* or condition* or discomfort* or pain* or distress* or burn* or fullness*) adj postprandial).tw.
6	(dyspep* adj (functional or non ulcer or nonulcer)).tw.
7	(indigestion adj (intestin* or digest* or gastr* or gut or epigastr* or stomach*)).tw.
8	(epigastric adj (pain* or burn*)).tw.
9	Rome criteria.tw.
10	((Gastroduodenal or gastrointestinal) adj symptom*).tw.
11	early satiety.tw.
12	((gastric or stomach or symptom*) adj acid adj secretion).tw.
13	1 or 2 or 3 or 4 or 5 or 6 or 7 or 8 or 9 or 10 or 11 or 12
14	Dietary Fats, Unsaturated/ or Dietary Fats/ or Fats, Unsaturated/ or Fats/
15	exp Carbohydrates/
16	exp Dietary Fiber/
17	Proteins/
18	exp Seafood/
19	Nuts/
20	exp Soy Foods/
21	Albumins/
22	Globulins/
23	glutens/ or gliadin/
24	Salicylates/
25	Amines/
26	exp Alcoholic Beverages/
27	Fruit/
28	Vegetables/
29	Edible Grain/
30	exp Meat/
31	exp Dairy Products/
32	Snacks/
33	Probiotics/
34	(saturated fat* or unsaturated fat* or carbohydrate* or fibre or fiber or fodmap* or sugar or protein* or food allergen* or fish or seafood* or egg* or nuts or soy or wheat protein* or albumin or globulin or gliadin or gluten* or salicylate* or amine or glutamate* or alcohol* or beer* or wine* or spirits or fruit* or vegetable* or grain* or meat or dairy or probiotic*).tw.
35	((snack* or treat* or junk or discretionary) adj5 food*).tw.
36	14 or 15 or 16 or 17 or 18 or 19 or 20 or 21 or 22 or 23 or 24 or 25 or 26 or 27 or 28 or 29 or 30 or 31 or 32

	or 33 or 34 or 35
37	((assess* or measure* or survey* or questionnaire* or record* or diary* or tool*) adj (diet* or food*)).tw.
38	((assess* or measure* or tool*) adj (nutrition* or nutrient*)).tw
39	Food frequency questionnaire/ or FFQ/
40	Food diary/
41	Weighed food record/ or WFR/
42	37 or 38 or 39 or 40 or 41
43	13 and 36 and 42
**44**	**limit 43 to (english language and yr="2000 -Current" and ("all adult (18 plus years)" or**
